# The γ-secretase inhibitors enhance the anti-leukemic activity of ibrutinib in B-CLL cells

**DOI:** 10.18632/oncotarget.19494

**Published:** 2017-07-22

**Authors:** Paola Secchiero, Rebecca Voltan, Erika Rimondi, Elisabetta Melloni, Emmanouil Athanasakis, Veronica Tisato, Stefania Gallo, Gian Matteo Rigolin, Giorgio Zauli

**Affiliations:** ^1^ Department of Morphology, Surgery and Experimental Medicine and LTTA Centre, University of Ferrara, Ferrara, Italy; ^2^ Institute for Maternal and Child Health, IRCCS “Burlo Garofolo”, Trieste, Italy; ^3^ Department of Medical Sciences, Section of Hematology, University of Ferrara, Ferrara, Italy

**Keywords:** B-leukemic cells, Ibrutinib, γ-secretase inhibitors, NOTCH1, combination therapy

## Abstract

Ibrutinib blocks B-cell receptor signaling and interferes with leukemic cell-to-microenvironment interactions. Ibrutinib plays a key role in the management of B-CLL and is recommended for first line treatment of high-risk CLL patients with 17p deletion. Therefore, elucidating the factors governing sensitivity/resistance to Ibrutinib represents a relevant issue. For this purpose, in 3 B-CLL patient samples harboring functional *TP53* mutations, the frequency of the mutated clones was monitored during *in vivo* Ibrutinib therapy, revealing a progressive decline of the frequency of *TP53^mut^* clones during 12 months of treatment. In parallel, the anti-leukemic activity of Ibrutinib was assessed *in vitro* on B-CLL patient cell cultures in combination with γ-secretase inhibitors (GSI). In the *in vitro* assays, the combination of Ibrutinib+GSI exhibited enhanced cytotoxicity on B-CLL cells also in the presence of stroma and it was coupled to the down-regulation of the stroma-activated NOTCH1 and c-MYC pathways. Moreover, the combined treatment was effective in reducing CXCR4 expression and functions. Therefore, the ability of GSI to enhance the Ibrutinib anti-leukemic activity in B-CLL cells, by down-regulating the NOTCH1 and c-MYC pathways, warrants further experimentation for its potential therapeutic applications.

## INTRODUCTION

The therapy of B chronic lymphocytic leukemia (B-CLL) is rapidly evolving, as inhibitors of B-cell receptor (BCR) signaling have shown substantial activity in the absence of traditional immune-chemotherapy [1, 2]. Among the inhibitors of BCR, Ibrutinib was the first described inhibitor of the Bruton tyrosine kinase (BTK) that irreversibly inhibits the BTK kinase through covalent binding [3]. Recently, the FDA approval for Ibrutinib has been extended to CLL patients regardless of their treatment history (treatment-naïve and previously-treated patients) and Ibrutinib is now recommended by the National Comprehensive Cancer Network (NCCN) for first line treatment of frail CLL patients with significant comorbidities, as well as for high-risk CLL patients with 17p deletion [3, 4]. Although the vast majority of B-CLL patients treated with Ibrutinib shows evident clinical benefit [4–7], some B-CLL patients develop progressive disease after prolonged treatment. Such secondary resistance to Ibrutinib has been associated, in some cases, with acquired mutations in either BTK or in its downstream target PLCγ [8, 9]. Recently it has been reported that the *TP53* mutation/17pdel combination, which represents a major determinant of resistance to immune-chemotherapy in B-CLL [10], might negatively interferes also with Ibrutinib efficacy [11, 12]. Regardless of the mutational status, the activation of NOTCH1 signaling, through interactions with its surface ligands, might render B-CLL cells more resistant to spontaneous and chemotherapy-induced apoptosis [13–15]. Indeed, the binding to NOTCH1 ligands, belonging to the Jagged or Delta-like ligand (DLL) families, triggers multiple proteolytic cleavages of the NOTCH1 protein, the last of which is operated by the γ-secretase enzyme, causing nuclear translocation of the intra-cellular domain of NOTCH1 (ICN) [16, 17].

Therefore, in order to start to elucidate the factors governing sensitivity/resistance to Ibrutinib, we sought to analyze: i) the clonal evolution of *TP53* mutations in a pilot group of B-CLL patients undergoing Ibrutinib therapy in a 12 months follow-up; ii) the potential anti-leukemic activity of the combination of Ibrutinib with γ-secretase inhibitors (GSI) by *in vitro* assays performed using B-CLL primary cells.

## RESULTS

### *In vivo* evolution of the frequency of *TP53^mut^* clones in response to ibrutinib therapy in a small subset of B-CLL patients

**Figure 1:**
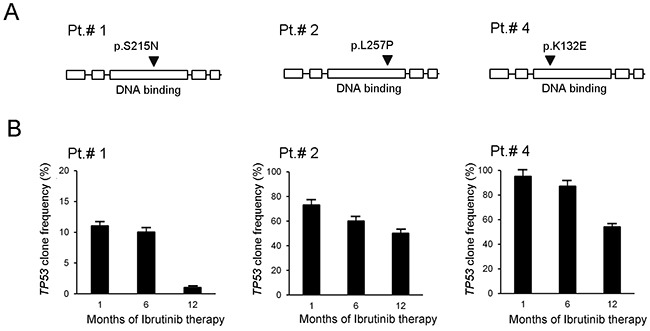
*In vivo* evolution of frequency of *TP53^mut^* clones in response to ibrutinib Longitudinal follow-up of clone frequency relatively to *TP53* analyzed by NGS on B-CLL primary cells of 3 patients treated *in vivo* with Ibrutinib therapy and in partial remission at the time of the 12^th^ -month of follow-up. In **(A)**, schematic representations of P53 protein domains (from left the boxes represent: transactivation domain, proline-rich domain, DNA-binding domain, tetramerization domain and regulatory domain) show the relative mutation site on the protein (arrowhead) characteristic of each patient. As shown, all mutations map in the DNA-binding domain that is responsible for DNA binding and target gene transactivation. In **(B)**, results of the *TP53^mut^* clone frequency follow-up are reported for each patient as mean±SD of analyses performed in triplicates.

For the present study, we analyzed a B-CLL population of 30 patients at different disease stage and characterized by different canonical clinical prognostic markers (CD38, IgHV status, chromosomal aberrations and *TP53* mutations) (Table 1). Among the B-CLL population analyzed, all characterized for having unmutated *BTK* and *PLCγ2*, six patients underwent to Ibrutinib therapy and three of them carried *TP53* functional mutations, in different genetic sites and at different clonal frequency (Table 1 and Figure 1). For these patients we could perform *TP53* analysis at different time points after Ibrutinib therapy. As reported in Figure 1, the *TP53*-clonal frequency of Pt.#1 declined from 11% to 1% after 12 months of Ibrutinib therapy. Similar trend was observed also in Pt.#2 (from 73% to 50%) and in Pt.#4 (from 95% to 54%) after 12 months of Ibrutinib therapy (Figure 1). These findings reinforce the notion of the efficacy of Ibrutinib in B-CLL carrying *TP53* mutations [18, 19], and provide the first evidence concerning the ability of Ibrutinib to target the *TP53^mut^* clones.

**Table 1:** Clinical and laboratory characteristics of B-CLL patients at the moment of *in vitro* treatment with ibrutinib

Pt. #	Age (years)/ gender	CD38+/ ZAP70+	IgHV status	Cytogenetic abnormalities	*TP53* mutation (clone frequency)	Therapy
**1**	74/M	pos/na	unmut	11qdel, 13qdel	c.644G>A (8%)	Ibrutinib
**2**	70/M	neg/neg	unmut	11qdel, 17pdel, 13qdel	c.770T>C (60%)	Ibrutinib
**3**	76/M	pos/na	na	na	c.380C>T (39.6%)c.920-2A>G(26.2%)	None
**4**	63/F	neg/neg	unmut	11qdel, 13qdel	c.394A>C (90%)	Ibrutinib
**5**	58/M	neg/neg	unmut	neg	unmu*t*	None
**6**	83/F	pos/neg	mut	13qdel, Trisomy 12	unmut	Steroid
**7**	71/M	neg/neg	mut	13qdel	unmut	None
**8**	69/M	neg/neg	mut	neg	unmut	None
**9**	56/M	pos/na	na	11qdel	unmut	Rituximab+ Ibrutinib
**10**	68/M	neg/neg	unmut	11qdel	unmut	Ibrutinib
**11**	58/F	pos/pos	unmut	13qdel	unmut	None
**12**	57/F	pos/neg	unmut	neg	unmut	Chl
**13**	59/M	neg/pos	unmut	neg	unmut	FCL
**14**	76/M	neg/neg	mut	13qdel	unmut	R-Benda
**15**	57/F	neg/neg	mut	13qdel	unmut	None
**16**	83/M	pos/neg	mut	13qdel	unmut	None
**17**	74/M	neg/pos	mut	13qdel	unmut	None
**18**	70/M	neg/neg	mut	13qdel	unmut	None
**19**	82/F	neg/neg	mut	Trisomy 12	unmut	None
**20**	76/M	pos/neg	unmut	13qdel	unmut	R-Benda
**21**	75/M	neg/neg	mut	neg	unmut	None
**22**	71/F	neg/neg	mut	13qdel	unmut	None
**23**	84/F	neg/neg	mut	13qdel	unmut	na
**24**	77/F	neg/neg	mut	Trisomy 12	unmut	None
**25**	85/M	neg/na	na	neg	unmut	None
**26**	36/F	pos/na	unmut	neg	unmut	FCR
**27**	65/M	neg/pos	mut	neg	unmut	None
**28**	47/M	pos/na	na	Trisomy 12	unmut	None
**29**	66/M	neg/neg	unmut	13qdel	unmut	Ibrutinib
**30**	68/F	pos/na	na	na	unmut	None

Pt., patient; R-Benda, rituximab-bendamustine; Chl, chlorambucil; FCR, fludarabine-cyclophosphamide-rituximab; FCL, fludarabine-cyclophosphamide-lenalidomide; neg, negative; pos, positive; del, deletion; na, not available; mut, mutated; unmut, unmutated

### *In vitro* cytotoxic effect of ibrutinib+GSI combination in B-CLL cells

**Figure 2:**
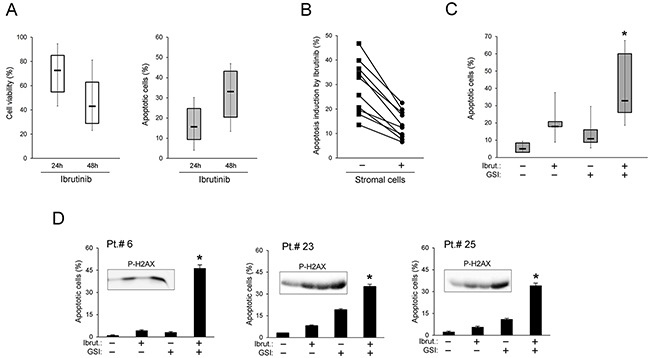
*In vitro* cytotoxic effect of Ibrutinib+GSI combination in primary B-CLL cell cultures Patients’ derived B-CLL cells were exposed *in vitro* to Ibrutinib±GSI for 24/48 hours. In **(A)**, cell viability, calculated as percentage with respect to the control untreated cultures (set to 100%), and apoptosis induction in response to Ibrutinib are shown. In **(B)**, apoptosis induction by Ibrutinib was comparatively assessed in primary B-CLL cells either kept in suspension or co-cultured with stromal cells. In the graph each line connects results of single patients. In **(C)** and **(D)**, cytotoxic effect of Ibrutinib±GSI, assessed in primary B-CLL cells co-cultured with stromal cells, was comparatively evaluated as induction of apoptosis. In C, results performed on B-CLL from 12-15 patients are shown. In D, results of cell cultures from representative patients are reported as mean±SD of three independent experiments and are shown together with P-H2AX levels analyzed by Western blotting (inserts). In A and C, horizontal bars are median, upper and lower edges of box are 75^th^ and 25^th^ percentiles, lines extending from box are 10^th^ and 90^th^ percentiles. Apoptosis induction was calculated as percentage of Annexin V/PI double positive cells. The asterisk indicates p<0.05 with respect to the single compound.

Cell cultures obtained from the same cohort of B-CLL patients (Table 1) were exposed *in vitro* to Ibrutinib, used at the concentration corresponding to the IC_50_ mean value determined in previous studies of our group in primary B-CLL cultures [11] and in line with other groups [20–23]. As shown in Figure 2A, *in vitro* treatment with Ibrutinib revealed a progressive reduction of cell viability coupled to the induction of apoptosis, with mean±SD (percentage of apoptotic cells over basal levels) of 18±12 and 32±15 at 24 and 48 hours of treatment, respectively. In particular, the *in vitro* response to Ibrutinib at 48 hours of treatment was comparable in B cell samples obtained from naïve B-CLL patients (mean±SD: 26±15) with respect to the patients under therapy with Ibrutinib and/or with chemo-immunotherapy (mean±SD: 35±13). Moreover, patient samples carrying *TP53* mutations showed a susceptibility to Ibrutinib cytotoxicity comparable to unmutated patient samples. These *in vitro* data are therefore in line with the *in vivo* data illustrated above.

For most patient samples, B-CLL cells were treated with Ibrutinib also in co-culture with stromal cells, mimicking the microenvironment of lymph node niches. As shown in Figure 2B, under co-culture conditions the response to Ibrutinib-cytotoxicity was reduced with respect to suspension B-CLL cultures, consistently with the protective role of B-CLL/stroma interactions against anti-leukemic drugs [24, 25]. On the other hand, the anti-leukemic cytotoxicity of Ibrutinib was enhanced by the combination with γ-secretase inhibitors (GSI, both PF-03084014 and L-685,458), as evaluated in terms of apoptosis and of P-H2AX levels (Figure 2C-2D). This effect was more evident in the B-CLL/stroma co-cultures than in suspension (Supplementary Figure 1) due to the lower toxicity of the treatment with the single drugs.

### Down-modulation of NOTCH1 and c-MYC pathways by ibrutinib±GSI in B-CLL cells

**Figure 3:**
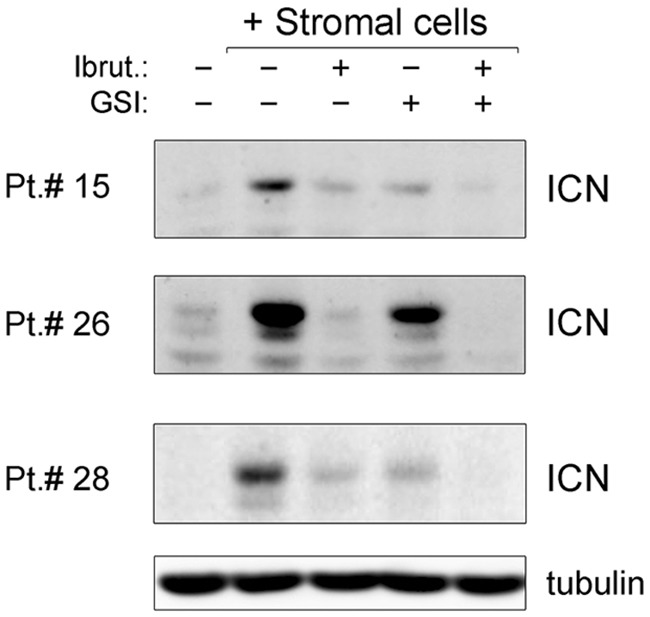
Down-modulation of NOTCH1 pathway by ibrutinib±GSI in primary B-CLL cell cultures Patients’ derived B-CLL cells co-cultured with stromal cells were exposed to Ibrutinib±GSI for 24 hours or were cultured untreated in suspension as control. Western blotting analyses of cleaved intracellular NOTCH1 (ICN) are shown for representative primary B-CLL patients. For clarity, tubulin is shown as loading control for one patient.

Since attachment of B-CLL cells to the stroma is known to protect B-CLL cells through the activation of different pro-survival pathways, including the NOTCH1 pathway [24, 25], in the next experiments the levels of NOTCH1 activation were analyzed by Western blotting. As expected, a marked NOTCH1 activation was observed in the B-CLL cells upon stromal co-culture, as documented by higher levels of the NOTCH1 intracellular domain (ICN) with respect to the untreated suspension culture, which were reduced by treatment with GSI (Figure 3). Unexpectedly, in both the culture settings (suspension and co-culture) also exposure to Ibrutinib alone down-regulated NOTCH1 activation, and the effect was enhanced when used in combination with GSI (Figure 3 and Supplementary Figure 2).

**Figure 4:**
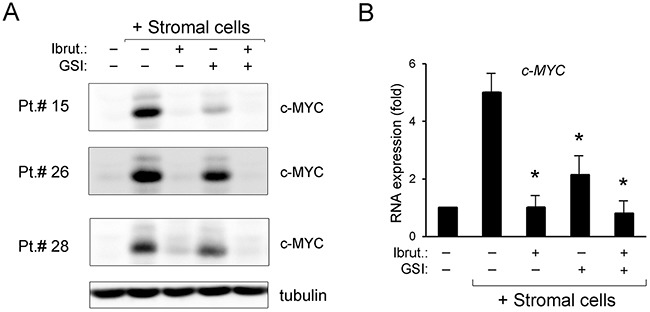
Down-regulation of c-MYC pathway by ibrutinib±GSI in primary B-CLL cell cultures Patients’ derived B-CLL cells co-cultured with stromal cells were exposed to Ibrutinib±GSI for 24 hours or were grown untreated in suspension as control. In **(A)**, Western blotting analyses of c-MYC protein levels are shown for representative primary B-CLL patients. For clarity, tubulin is shown as loading control for one patient. In **(B)**, levels of *c-MYC* mRNA were analyzed by qRT-PCR and are expressed as fold of modulation with respect to the untreated B-CLL cultures grown in suspension set at 1. Results are reported as mean±SD of four independent experiments, performed in duplicate. The asterisk indicates p<0.05 with respect to untreated B-CLL cells co-cultured with stromal cells.

In parallel, we have analyzed the oncogenic transcription factor c-MYC expression, which has recently been shown to be a target of both BTK [26, 27] and NOTCH1 [24, 25] pathways in different hematological malignancies, including B-CLL. Moreover, over-expression of c-MYC is involved in B cell transformation [28] and has been linked to potential Ibrutinib resistance [27]. As shown in Figure 4, under co-culture conditions a significant induction of c-MYC was documented in B-CLL samples both by protein and mRNA expression analysis. Of note, both GSI and, even more, Ibrutinib, either alone or in combination, counteracted the upregulation of c-MYC induced by the contact with stroma (Figure 4A-4B). Although the baseline *c-MYC* mRNA levels were significantly lower in B-CLL cells suspension, respect to the same samples co-cultured with stroma (Figure 4B), a significant down-modulation induced by the combination was observed also in this setting (Supplementary Figure 3).

### Effect of ibrutinib±GSI on CXCR4/SDF-1α-mediated migration of B-CLL cells

**Figure 5:**
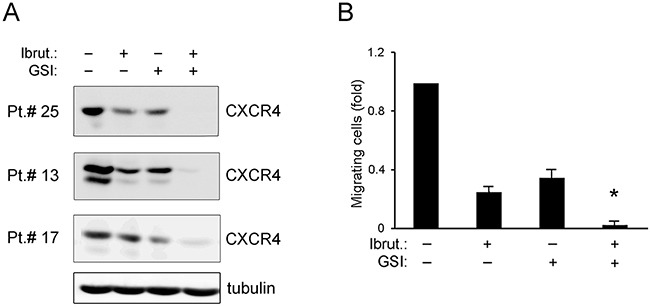
Effect of ibrutinib±GSI on CXCR4/SDF-1α-mediated migration of primary B-CLL cell cultures Patients’ derived B-CLL cells were exposed *in vitro* to Ibrutinib±GSI for 24 hours. In **(A)**, Western blotting analyses of CXCR4 total protein levels are shown for representative primary B-CLL cultures. For clarity, tubulin is shown as loading control for one patient. In **(B)**, the number of migrated B-CLL cells towards SDF-1α is expressed as fold with respect to control untreated cultures set at 1. Results are reported as mean±SD of four independent experiments. The asterisk indicates p<0.05 with respect to single treatments.

CXCR4 has been identified as a key regulator in CLL-cell retention in bone marrow and lymphoid tissues and it has recently been shown that BTK inhibition impairs CXCR4 expression in B-CLL [29]. In addition, in the multiple myeloma setting a direct positive control of CXCR4 by NOTCH1 has been recently proposed [30]. On these bases, in the last series of experiments we have assessed the effect of Ibrutinib, alone or in combination with GSI, on CXCR4 expression in B-CLL primary cells. As shown in Figure 5A, the exposure to Ibrutinib, as well as to GSI, significantly decreased the levels of CXCR4. The functional relevance of the CXCR4 down-regulation induced by Ibrutinib, GSI and combination thereof, was assessed by migration assays (Figure 5B). As shown in Figure 5B, the leukemic cell migration in response to recombinant human SDF-1α (10 ng/ml) was significantly impaired by pretreatment with Ibrutinib and GSI used alone, and completely abrogated by the drug combination.

## DISCUSSION

Summarizing the major findings of our study, we found that B-CLL samples with *TP53* mutations showed sensitivity to Ibrutinib treatment, as suggested by both *in vivo* and *in vitro* analyses. Indeed, in the pilot group of patients under treatment with Ibrutinib for 12 months we documented a progressive decrease of the percentages of *TP53^mut^* clones. Moreover, the ability of Ibrutinib to induce apoptosis *in vitro* in B-CLL patient cultures carrying *TP53* mutations was similar to unmutated B-CLL samples.

The *in vitro* experiments performed by treating primary B-CLL cultures with Ibrutinib used in combination with GSI showed enhanced B-CLL cell apoptosis both in the absence or presence of stroma, a condition mimicking the B-CLL microenvironment. Although the concentrations of Ibrutinib used in our *in vitro* assays exceeded the values found in the plasma of patients treated *in vivo* with Ibrutinib [21], these doses were necessary to evaluate the anti-leukemic effect in different biological assays and comparatively among different B-CLL samples. In this respect, the results obtained in the co-culture setting are particularly relevant since it is well established that the stromal niche exerts a protective effect on CLL cells also attenuating their drug sensitivity [24]. Of note, at the molecular level, the *in vitro* treatment with Ibrutinib down-regulated: *i*) the pro-survival NOTCH1 pathway, which represents a major molecular target of GSI [31] and plays a relevant role in B-CLL leukemogenesis [32]; *ii*) the c-MYC expression, which is constitutively activated *in situ* in the lymph nodes of B-CLL patients [33], and has been proposed to confer resistance to Ibrutinib in B-NHL [27]; *iii*) CXCR4 expression, which plays a key role in the retention of B-CLL into the microenvironmental niches [34], coupled to reduction of migratory response of B-CLL cells to SDF-1α. The effects of Ibrutinib on the NOTCH1 and c-MYC pathways, as well as on CXCR4/SDF-1α were enhanced by the combination with GSI.

Overall, our findings are of particular relevance since *in vivo* the B-CLL cells in secondary lymphoid organs are in contact with several cell types and secreted molecules that together constitute the B-CLL microenvironment [35]. It has been demonstrated that leukemia cell-to-microenvironment interactions are weakened upon exposure to Ibrutinib, and a fraction of B-CLL cells egresses the lymphoid organs and relocates to the blood stream [36]. Thus, it is particularly noteworthy that Ibrutinib+GSI combination promoted enhanced cytotoxicity also on B-CLL cells co-cultured in the presence of stromal cells.

Another important issue is represented by the potential clinical relevance of our data with respect to the feasibility of using the Ibrutinib+GSI combination for the treatment of B-CLL patients. In this respect, it should be noticed that a number of GSI have been clinically evaluated as anti-cancer agents, including semagacestat (LY450139), RO4929097, avagacestat (BMS-708163), PF-03084014 and 3-[(1r, 4s)-4-(4-chloroph enylsulfonyl)-4-(2,5-difluorophenyl) cyclohexyl] propanoic acid (MK-0752) (data concerning these trials are available at clinicaltrials.gov). In spite of initial disappointing results [31], recent phase I clinical trials with the novel GSI PF-03084014, used for our *in vitro* assays, documented a complete response and several partial responses in advanced cancers [37]. Besides application of GSI as single agent, a wide number of combination studies incorporating GSI with established anti-cancer treatments have been evaluated and/or are currently undergoing evaluation for therapeutic efficacy (details are available from clinicaltrials.gov), including drugs with different molecular targets: capecitabine, a fluorouracil prodrug; bicalutamide, an androgen antagonist; letrozole, a nonsteroidal aromatase inhibitor; temozolomide, an alkylating agent; tamoxifen, an antiestrogen; erlotinib hydrochloride, an inhibitor of epidermal growth factor receptor tyrosine kinase; gemcitabine hydrochloride, an antimetabolite; vinblastine and docetaxel, microtubule targeting agents; cisplatin, a DNA targeting agent; and cediranib maleate, a vascular endothelial growth factor receptor-2 tyrosine kinase inhibitor. Moreover, of particular relevance for the aim of this study, promising results have been obtained in multiple myeloma, by employing GSI in combination with proteasome inhibitors [38], as well as in B-CLL by using GSI in association with conventional chemotherapy [39, 40]. Finally, it is also noteworthy that novel pan-NOTCH GSI have been recently described [41, 42].

In this context, we have demonstrated for the first time that the GSI enhanced the cytotoxicity induced by Ibrutinb in B-CLL both in the absence and presence of stromal cells, by down-modulating the NOTCH1 and the c-MYC pathways. In addition, the Ibrutinib+GSI combination down-regulated the level of CXCR4 in B-CLL and, subsequently, migratory ability towards SDF-1α. Considering that combination therapies with Ibrutinib are advancing into the clinic, our data provide a rationale for the use of novel Ibrutinib-based combinations with GSI to overcome stroma/NOTCH1-mediated drug resistance.

## MATERIALS AND METHODS

### B-CLL patient samples collection

Peripheral blood samples were collected in heparin coated tubes, following informed consent, in accordance with the Declaration of Helsinki and in agreement with institutional guidelines (University-Hospital of Ferrara), from a cohort of 30 B-CLL patients. The clinical, laboratory and cytogenetic data (CD38 and ZAP70 surface expression, FISH and IgHV status) of each patient were abstracted from clinical and laboratory records. *TP53*, *BTK* and *PLCγ2* mutations were analyzed by NGS as previously described [43]. To identify pathogenic variations, mutations that did not affect the protein coding regions (intronic, 3’ and 5’ UTR variations, silent exonic mutations and polymorphisms) were filtered out.

All patients have been without prior therapy at least for three weeks before peripheral blood collection. Peripheral blood mononuclear cells (PBMC) were isolated from B-CLL patient's by gradient centrifugation with lymphocyte cell separation medium (Cedarlane Laboratories, Hornby, ON). T lymphocytes, NK lymphocytes, granulocytes and monocytes were negatively depleted from B-CLL PBMC with immunomagnetic microbeads (MACS microbeads, Miltenyi Biotech, Auburn, CA), with a purity >95% of resulting CD19^+^ population. Freshly isolated primary cells were cultured in RPMI-1640 medium containing 10% FBS, L-glutamine and penicillin/streptomycin (Gibco, Grand Island, NY), or conserved within Hematopathology collection of our institution (Department of Morphology, Surgery and Experimental Medicine and LTTA Centre, University of Ferrara). For co-culture experiments, primary B-CLL cells were added to a sub-confluent monolayer of stromal cells and cultured in complete RPMI-1640 medium (Gibco) at 37°C in a humidified atmosphere containing 5% CO_2_.

### Culture treatments and assessment of cell viability and apoptosis

For *in vitro* treatments of cell cultures, Ibrutinib (PCI-32765; Selleckchem, Houston, TX) was used either alone or in combination with GSI (PF-03084014 or L-685,458; Sigma-Aldrich, St. Louis, MO). B-CLL cells were seeded, either alone or in co-culture with stromal cells, at a density of 2×10^6^ cells/mL. Optimal concentrations for the compounds (1-10 μM each) were determined in dose-response assays. At different time points after treatment, cell viability was examined by Trypan blue dye exclusion and MTT (3-(4,5-dimethilthiazol-2yl)-2,5-diphenyl tetrazolium bromide) colorimetric assay (Roche Diagnostics Corporation, Indianapolis, IN) for data confirmation, as previously described [44]. The amount of apoptosis was quantified by Annexin V-FITC/propidium iodide (PI) staining (Beckman Coulter Inc., Brea, CA) using a FACSCalibur flow cytometer (BD Biosciences, San Josè, CA). To avoid non-specific fluorescence from dead cells, live cells were gated tightly using forward and side scatter, as described [45].

### RNA and protein analyses

Total RNA was extracted from cells using the QIAGEN miRNeasy Mini kit (QIAGEN, Hilden, Germany), accordingly to the supplier's instructions. Genomic DNA was removed with RNase-Free DNase set. For each sample, total RNA (300 ng) was transcribed into cDNA and amplified using the Express One-Step Superscript qRT-PCR Kit, universal (Thermo Fisher Scientific, Rockford, IL). Analysis of human *c-MYC* expression was carried out with validated TaqMan Gene Expression Assays specific PCR primers sets (Thermo Fisher Scientific). All samples were run in duplicate using the real time thermal analyzer Applied Biosystems 7500 Fast Real-Time PCR System (Thermo Fisher Scientific). Expression values were normalized to the housekeeping gene *POLR2A* amplified in the same sample.

For Western blotting analysis, cells were lysed as previously described [46]. Protein determination was performed by BCA Protein Assay (Thermo Scientific). Equal amounts of proteins for each sample were migrated in SDS-polyacrylamide gels and blotted onto nitrocellulose filters. The following Abs were used: anti cleaved NOTCH1 (Val1744) (D3B8), anti CXCR4 (D4Z7W), anti c-MYC (D3N8F) and anti phospho-histone H2A.X (Ser139), all from Cell Signaling (Danvers, MA); anti tubulin, from Sigma-Aldrich. After incubation with anti-mouse or anti-rabbit IgG horseradish peroxidase-conjugated secondary antibodies (Sigma-Aldrich), specific reactions were revealed with the ECL Lightning detection kit (Perkin Elmer, Waltham, MA). Images acquisition was performed using the ImageQuant™ LAS 4000 biomolecular imager (GE Healthcare, Buckinghamshire, UK).

### Migration assays

Migration assays were performed in trans-well plates (Corning Costar, Cambridge, MA) 6.5 mm in diameter, with 5-μm pore filters, as described [47]. Briefly, CD19^+^ cells derived from B-CLL patients either left untreated or pretreated with Ibrutinib and GSI, alone or in combination, were added to the upper chamber, while SDF-1α (10 ng/ml, R&D Systems, Minneapolis, MN) was added to the lower chamber as chemoattractant. After 3 hours of incubation at 37°C in 5% CO_2_, the upper side of the filters was removed and cells migrated to the lower chamber were counted using a FACSCalibur flow cytometer (BD Biosciences). Each experiment was done in duplicate.

### Statistical analysis

Statistical analysis data were calculated as median or mean±SD. Box plots were used to show the median and interquartile values for each group of data. The results were evaluated by using an ANOVA with subsequent comparisons by a Student's t-test and with the MannWhitney rank-sum test. Statistical significance was defined as p<0.05.

## SUPPLEMENTARY MATERIALS FIGURES AND TABLES



## Abbreviations

BCR: B-cell receptor
B-NHL: B-cell Non-Hodgkin lymphoma
BTK: Bruton's tyrosine kinase
CD: cluster of differentiation
Chl: chlorambucil
CLL: chronic lymphocytic leukemia
CXCR4: C-X-C chemokine receptor type 4
FCL: fludarabine-cyclophosphamide-lenalidomide
FCR: fludarabine-cyclophosphamide-rituximab
FDA: Food and Drug Administration
FISH: fluorescent in situ hybridization
H2AX: Histone Family Member X
ICN: intra-cellular domain of NOTCH1
IgHV: immunoglobulin heavy-chain variable region
GSI: γ-secretase inhibitors
MTT: 3-(4,5-dimethilthiazol-2yl)-2,5-diphenyl tetrazolium bromide
NCCN: National Comprehensive Cancer Network
NGS: Next Generation Sequencing
PBMC: Peripheral blood mononuclear cells
PLCγ: phospholipase C-gamma
R-Benda: rituximab-bendamustine
SDF-1alpha: stromal cell-derived factor 1α
ZAP70: 70 kDa zeta-associated protein
