# Supplementary material for: The γ-secretase inhibitors enhance the anti-leukemic activity of ibrutinib in B-CLL cells
Source: Oncotarget. 2017 Jul 22;8(35):59235–45. doi: 10.18632/oncotarget.19494 (PMC5601728; doi:10.18632/oncotarget.19494)
Supplement: Supplementary file 1 [file oncotarget-08-59235-s001.pdf]

The γ-secretase inhibitors enhance the anti-leukemic activity of ibrutinib in B-CLL cells

SUPPLEMENTARY MATERIALS

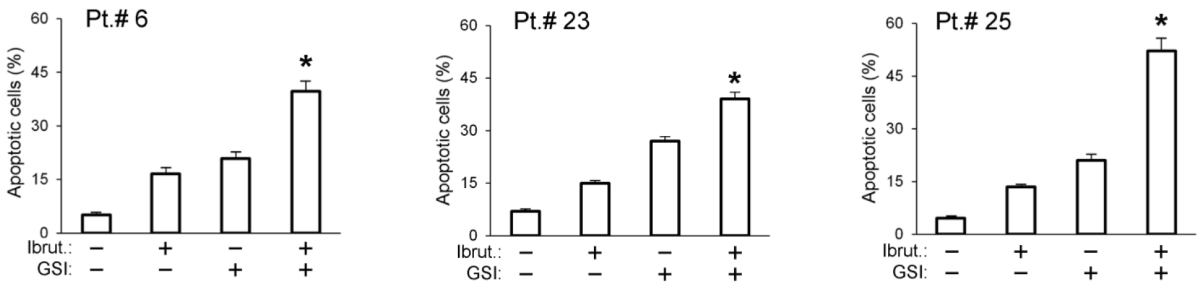

Supplementary Figure 1: *In vitro* cytotoxic effect of ibrutinib+GSI combination in primary B-CLL cells cultured in suspension. Patients' derived B-CLL cells, cultured in suspension, were exposed *in vitro* to Ibrutinib±GSI for 24/48 hours. Cytotoxic effect was evaluated as induction of apoptosis calculated as percentage of Annexin V/PI double positive cells. Results of cell cultures from representative patients are shown and are reported as mean±SD of three independent experiments. The asterisk indicates p<0.05 with respect to the single compound.

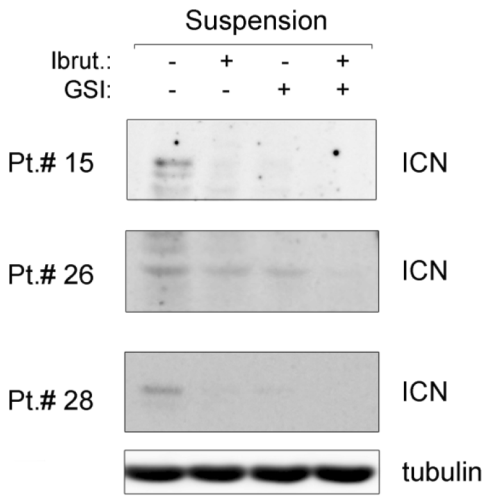

Supplementary Figure 2: Down regulation of NOTCH1 pathway by ibrutinib±GSI in primary B-CLL cells. Patients' derived B-CLL cells cultured in suspension were exposed to Ibrutinib±GSI for 24 hours. Western blotting analyses of cleaved intracellular NOTCH1 (ICN) protein levels are shown after long exposure for representative primary B-CLL patients. For clarity, tubulin is shown as loading control for one patient.

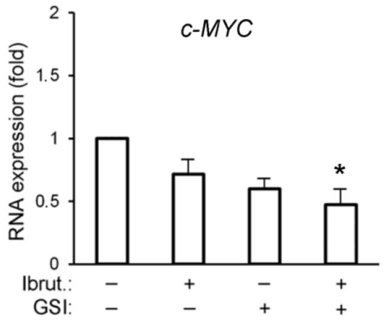

Supplementary Figure 3: Down regulation of c-MYC pathway by ibrutinib±GSI in primary B-CLL cells cultured in suspension. Patients' derived B-CLL cells, cultured in suspension, were exposed to Ibrutinib±GSI for 24 hours. Levels of *c-MYC* mRNA were analyzed by quantitative RT-PCR and are expressed as fold of modulation with respect to the control untreated cultures set at 1. Results are reported as mean±SD of three independent experiments, performed in duplicate. The asterisk indicate $p<0.05$ with respect to the untreated.
